# Association of Initial SARS-CoV-2 Test Positivity With Patient-Reported Well-being 3 Months After a Symptomatic Illness

**DOI:** 10.1001/jamanetworkopen.2022.44486

**Published:** 2022-12-01

**Authors:** Lauren E. Wisk, Michael A. Gottlieb, Erica S. Spatz, Huihui Yu, Ralph C. Wang, Benjamin H. Slovis, Sharon Saydah, Ian D. Plumb, Kelli N. O’Laughlin, Juan Carlos C. Montoy, Samuel A. McDonald, Zhenqiu Lin, Jin-Mann S. Lin, Katherine Koo, Ahamed H. Idris, Ryan M. Huebinger, Mandy J. Hill, Nicole L. Gentile, Anna Marie Chang, Jill Anderson, Bala Hota, Arjun K. Venkatesh, Robert A. Weinstein, Joann G. Elmore, Graham Nichol

**Affiliations:** 1 Division of General Internal Medicine and Health Services Research, David Geffen School of Medicine at the University of California, Los Angeles, Los Angeles; 2 Department of Health Policy and Management, Fielding School of Public Health at the University of California, Los Angeles, Los Angeles; 3 Department of Emergency Medicine, Rush University Medical Center, Chicago, Illinois; 4 Section of Cardiovascular Medicine, Department of Internal Medicine, Yale School of Medicine, New Haven, Connecticut; 5 Center for Outcomes Research and Evaluation, Yale New Haven Hospital, New Haven, Connecticut; 6 Department of Emergency Medicine, University of California, San Francisco, San Francisco; 7 Department of Emergency Medicine, Thomas Jefferson University, Philadelphia, Pennsylvania; 8 National Center for Immunization and Respiratory Diseases, Centers for Disease Control and Prevention, Atlanta, Georgia; 9 Department of Emergency Medicine, University of Washington, Seattle; 10 Department of Global Health, University of Washington, Seattle; 11 Department of Emergency Medicine, University of Texas Southwestern Medical Center, Dallas; 12 Clinical Informatics Center, University of Texas Southwestern Medical Center, Dallas; 13 National Center for Emerging and Zoonotic Infectious Diseases, Centers for Disease Control and Prevention, Atlanta, Georgia; 14 Department of Internal Medicine, Rush University Medical Center, Chicago, Illinois; 15 Department of Internal Medicine, University of Texas Southwestern Medical Center, Dallas; 16 Department of Emergency Medicine, McGovern Medical School, UTHealth Houston, Houston, Texas; 17 Department of Family Medicine, University of Washington, Seattle; 18 Department of Laboratory Medicine and Pathology, University of Washington, Seattle; 19 Department of Medicine, Harborview Center for Prehospital Emergency Care, University of Washington, Seattle; 20 Tendo Systems, Chicago, Illinois; 21 Department of Emergency Medicine, Yale School of Medicine, New Haven, Connecticut; 22 Department of Medicine, Rush University Medical Center, Chicago, Illinois; 23 Division of Infectious Diseases, Cook County Health, Chicago, Illinois

## Abstract

**Importance:**

Long-term sequelae after symptomatic SARS-CoV-2 infection may impact well-being, yet existing data primarily focus on discrete symptoms and/or health care use.

**Objective:**

To compare patient-reported outcomes of physical, mental, and social well-being among adults with symptomatic illness who received a positive vs negative test result for SARS-CoV-2 infection.

**Design, Setting, and Participants:**

This cohort study was a planned interim analysis of an ongoing multicenter prospective longitudinal registry study (the Innovative Support for Patients With SARS-CoV-2 Infections Registry [INSPIRE]). Participants were enrolled from December 11, 2020, to September 10, 2021, and comprised adults (aged ≥18 years) with acute symptoms suggestive of SARS-CoV-2 infection at the time of receipt of a SARS-CoV-2 test approved by the US Food and Drug Administration. The analysis included the first 1000 participants who completed baseline and 3-month follow-up surveys consisting of questions from the 29-item Patient-Reported Outcomes Measurement Information System (PROMIS-29; 7 subscales, including physical function, anxiety, depression, fatigue, social participation, sleep disturbance, and pain interference) and the PROMIS Short Form–Cognitive Function 8a scale, for which population-normed T scores were reported.

**Exposures:**

SARS-CoV-2 status (positive or negative test result) at enrollment.

**Main Outcomes and Measures:**

Mean PROMIS scores for participants with positive COVID-19 tests vs negative COVID-19 tests were compared descriptively and using multivariable regression analysis.

**Results:**

Among 1000 participants, 722 (72.2%) received a positive COVID-19 result and 278 (27.8%) received a negative result; 406 of 998 participants (40.7%) were aged 18 to 34 years, 644 of 972 (66.3%) were female, 833 of 984 (84.7%) were non-Hispanic, and 685 of 974 (70.3%) were White. A total of 282 of 712 participants (39.6%) in the COVID-19–positive group and 147 of 275 participants (53.5%) in the COVID-19–negative group reported persistently poor physical, mental, or social well-being at 3-month follow-up. After adjustment, improvements in well-being were statistically and clinically greater for participants in the COVID-19–positive group vs the COVID-19–negative group only for social participation (β = 3.32; 95% CI, 1.84-4.80; *P* < .001); changes in other well-being domains were not clinically different between groups. Improvements in well-being in the COVID-19–positive group were concentrated among participants aged 18 to 34 years (eg, social participation: β = 3.90; 95% CI, 1.75-6.05; *P* < .001) and those who presented for COVID-19 testing in an ambulatory setting (eg, social participation: β = 4.16; 95% CI, 2.12-6.20; *P* < .001).

**Conclusions and Relevance:**

In this study, participants in both the COVID-19–positive and COVID-19–negative groups reported persistently poor physical, mental, or social well-being at 3-month follow-up. Although some individuals had clinically meaningful improvements over time, many reported moderate to severe impairments in well-being 3 months later. These results highlight the importance of including a control group of participants with negative COVID-19 results for comparison when examining the sequelae of COVID-19.

## Key Points

**Question:**

How do patient-reported physical, mental, and social well-being compare at 3 months after symptomatic illness among those who tested positive vs negative for SARS-CoV-2 infection?

**Findings:**

In this cohort study of 1000 US adults with symptomatic illness, poor well-being scores at follow-up were common in both those who tested positive and negative for SARS-CoV-2 infection. Despite some improvements over time, 39.6% of COVID-19–positive and 53.5% of COVID-19–negative patients reported residual symptoms.

**Meaning:**

These findings emphasize the importance of including a concurrent control group when studying sequelae of COVID-19 illness.

## Teaser

This cohort study compares patient-reported physical, mental, and social well-being at 3 months after symptomatic illness among adults with positive vs negative test results for SARS-CoV-2 infection.

## Introduction

Post–COVID-19 conditions (PCCs), often referred to as long COVID, are a heterogeneous group of conditions generally referring to symptoms that emerge, recur, or persist for more than 4 weeks after acute infection with SARS-CoV-2.^1^ Hallmarks of PCCs include fatigue, cognitive impairment, and postexertional malaise along with symptoms encompassing nearly every organ system.^2^ Previous literature suggests that up to one-half of those with SARS-CoV-2 infection experience persistent symptoms more than 4 weeks after acute infection.^3,4,5,6^ Studies of PCCs have primarily described discrete symptoms and/or health care use,^3^ with few addressing patient-reported outcomes such as health-related quality of life.

Assessment of patient-reported outcomes after COVID-19 can provide a deeper understanding of the patient experience and the ways in which the pandemic has impacted physical, mental, and social well-being.^7^ Individuals who have experienced social isolation, work disruption, or hospitalization during the pandemic might report similar impairments in well-being without antecedent COVID-19 illness^8,9^; therefore, inclusion of a concurrent control group is important to fully understand how well-being progresses after COVID-19. Well-established tools for measuring well-being are the Patient-Reported Outcomes Measurement Information System (PROMIS) instruments. These scales were developed and validated for the evaluation of patient-centric health domains, such as pain, fatigue, physical functioning, sleep, and emotional distress, that have major consequences for quality of life.^10,11^ The PROMIS instruments provide data on a prepandemic population-standardized norm for these domains, which facilitates comparisons across groups and against prepandemic expected values.

The Innovative Support for Patients With SARS-CoV-2 Infections Registry (INSPIRE) study was designed to prospectively assess long-term outcomes of adults with symptomatic acute COVID-19 alongside contemporary controls comprising adults who had similar symptoms but tested negative for SARS-CoV-2.^12^ In this interim analysis involving the first 1000 participants, we describe the patient-reported outcomes of physical and mental well-being (measured by the 29-item PROMIS [PROMIS-29] survey, version 2.1) and cognitive functioning (measured by the PROMIS Short Form–Cognitive Function [PROMIS SF-CF] 8a survey) at baseline and 3-month follow-up among participants with symptomatic illness who tested positive for COVID-19 vs those with symptomatic illness who tested negative at initial enrollment. Individuals with symptomatic illness who tested negative for SARS-CoV-2 infection were selected as the comparator group to identify the consequences of infection specifically with SARS-CoV-2 (vs another virus) for changes in well-being.

## Methods

### Study Design and Data Source

INSPIRE is an ongoing multicenter prospective longitudinal registry study enrolling individuals with acute symptoms suggestive of COVID-19 in 8 sites across the US. Recruitment occurs in person, by phone or email, and through online advertisement. A secure online platform (Hugo; Hugo Health LLC) facilitates the collation of consent-related materials, linkage to participants’ electronic health records, and responses to self-administered surveys. This study involved self-enrollment via an online consent process using an electronic consent form implemented in the Hugo platform; all included participants provided electronic informed consent. This study was approved by the institutional review boards across 8 sites: Rush University (Chicago, Illinois), Yale University (New Haven, Connecticut), the University of Washington (Seattle), Thomas Jefferson University (Philadelphia, Pennsylvania), the University of Texas Southwestern Medical Center (Dallas), UTHealth Houston (Houston, Texas), the University of California, San Francisco (San Francisco), and the University of California, Los Angeles (Los Angeles). A detailed description of the study design has been published.^12^ This study followed the Strengthening the Reporting of Observational Studies in Epidemiology (STROBE) reporting guideline for cohort studies.

### Cohort Definition

This study included adult participants (aged ≥18 years) who were recently under clinical investigation for SARS-CoV-2 infection, were fluent in English or Spanish, had self-reported symptoms suggestive of acute SARS-CoV-2 infection (eAppendix in Supplement 1),^13^ and received testing for SARS-CoV-2 infection with any molecular or antigen-based assay approved or authorized by the US Food and Drug Administration within 42 days before enrollment. An individual was ineligible if the study team was unable to confirm the result of a diagnostic test for COVID-19 or if the individual was unable to provide informed consent, lacked access to an internet-enabled device or computer that would allow for participation, had a SARS-CoV-2 infection more than 42 days before enrolling in the study, or was imprisoned while participating in the study. The goal was to recruit individuals at a 3:1 ratio of those with positive results for COVID-19 to those with negative results for COVID-19.^12^

Participants were grouped based on their initial COVID-19 status (ie, COVID-19 positive or negative at enrollment). If more than 1 COVID-19 test was performed within 7 days of enrollment and results were discordant, we considered the positive test results to be the true measure. However, if participants’ test positivity changed during the study (ie, later than 7 days after enrollment), we retained them in their initial group following an intention-to-treat approach. In this cohort, 2 of 1000 participants (0.2%) converted from the COVID-19–negative group to the COVID-19–positive group during the initial 3-month study period.

**Figure 1.**
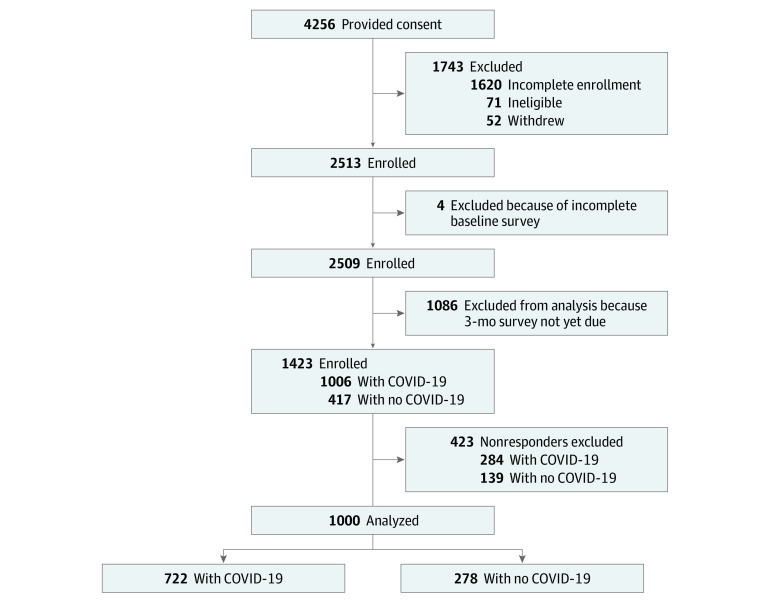
INSPIRE Participant Flow Diagram INSPIRE indicates Innovative Support for Patients With SARS-CoV-2 Infections Registry.

In this interim analysis, we included the first 1000 participants who completed the PROMIS surveys^12^ at baseline and 3-month follow-up (Figure 1). Participants included in this analysis were enrolled from December 11, 2020, to September 10, 2021.

### Cohort Characteristics

Participants self-reported sociodemographic data at baseline, including age, gender (female; male; or transgender, nonbinary, or other genders), ethnicity (Hispanic or non-Hispanic) or race (Asian, Native Hawaiian, or other Pacific Islander; Black or African American; White; other race; or more than 1 race), educational level (less than high school diploma, high school or general educational development diploma, some college but did not complete degree, 2-year college degree, 4-year college degree, or more than 4-year college degree), marital status (married or living with a partner; divorced, widowed, or separated; or never married), annual family income before the pandemic (<$10 000, $10 000-$35 000, $35 000-$50 000, $50 000-$75 000, or >$75 000), health insurance (private only, public only, private and public, or none), and employment status before the pandemic (employed as an essential worker or health care worker, employed as a nonessential worker or non–health care worker, or not employed). Participants also provided information on chronic conditions, location of their COVID-19 testing, and symptoms. Self-reports of symptoms suggestive of COVID-19 were assessed using questions derived from the Centers for Disease Control and Prevention Person Under Investigation for SARS-CoV-2 survey.^13^ Self-reported race and ethnicity data from the Person Under Investigation survey were included because SARS-CoV-2 infection, testing, and outcomes have been reported to vary across racial and ethnic groups.^14^

### Patient-Reported Outcomes

The baseline and 3-month surveys included questions from the PROMIS-29 and the PROMIS SF-CF 8a.^15^ The PROMIS instruments use T score measurement, in which a score of 50 represents the mean score of a reference population (ie, the US general population), with an SD of 10.^11,16^ For PROMIS measures, higher scores correspond to a greater degree of the outcome being measured (eg, greater fatigue). The T score was used to measure outcomes for 7 of the PROMIS-29 subscales (physical function, anxiety, depression, fatigue, social participation,^1^ sleep disturbance, and pain interference) and the PROMIS SF-CF 8a survey. A single-item numerical rating scale for pain intensity (“In the last 7 days, how would you rate your pain on average?”; score range, 0-10, with 0 indicating no pain and 10 indicating worst imaginable pain) is included in the PROMIS-29 survey. Due to a survey specification clerical error, participants were incorrectly presented with the 4 response options for the 9-item Public Health Questionnaire (ie, with 1 indicating not at all, 2 indicating several days, 3 indicating more than half of the days, and 4 indicating early every day) in place of the 5 response options for the PROMIS-29 subdomain measuring the ability to participate in social roles and activities (ie, with 1 indicating never, 2 indicating rarely, 3 indicating sometimes, 4 indicating usually, and 5 indicating always). Therefore, to calculate the scaled T score for the social participation domain, we conducted an equivalent score mapping correction after data collection, which resulted in 4 possible scores (ie, with 1 indicating never, 2.33 indicating rarely, 3.67 indicating sometimes or usually, and 5 indicating always).

In addition to reporting scaled T scores for these measures, we applied previously defined cutoffs to subscales to identify high levels of impairment.^17^ For physical function, social participation, and cognitive function (for which higher scores are better), scores lower than 40 represent moderate to severe impairment, and scores of 40 or higher represent normal to mild impairment. For anxiety, depression, fatigue, sleep disturbance, and pain interference (for which lower scores are better), scores lower than 60 represent normal to mild impairment, and scores of 60 or higher represent moderate to severe impairment.

The T score cutoff for a clinically meaningful within-group change generally ranges between 2 and 6 points.^18^ Therefore, we interpreted changes in T scores of at least 2 points to represent clinically meaningful changes in well-being measures across time points (with the exception of pain intensity, for which clinically meaningful changes were represented by T score changes of ≥1 point^19^).

### Statistical Analysis

Statistical analyses were performed using SAS software, version 9.4 (SAS Institute Inc). All tests were 2-sided with a significance threshold of *P* = .05. Sociodemographic and clinical characteristics of the COVID-19 groups (COVID positive vs COVID negative) were compared using χ^2^ tests for categorical variables and *t* tests for continuous variables. The frequency of missingness differed across participant characteristics and ranged from 2 to 63 missing values. No systematic patterns in missingness were observed; therefore, missingness at random was assumed. All percentages and *P* values were calculated after excluding missing values.

Scaled scores on PROMIS measures were compared by COVID-19 status at both baseline and 3-month follow-up using *t* tests, and categorical thresholds for PROMIS subscales (eg, normal to mild anxiety vs moderate to severe anxiety) were compared by COVID-19 status using χ^2^ tests. We evaluated bivariate change measures (difference in PROMIS scores between baseline and 3-month follow-up), comparing by COVID-19 status, using *t* tests. We used sequential multivariable linear regression analysis to model changes in PROMIS scores; the regression coefficient for the difference over time (baseline vs 3-month follow-up) by COVID-19 status (positive vs negative) was reported. First, unadjusted estimates were calculated, followed by models adjusting for demographic characteristics (including age and race), then social factors (including marital status, income, employment, and health insurance), then health conditions (including asthma, hypertension, and diabetes), then the baseline values of each PROMIS measure (eg, modeling change in physical function as the outcome, adjusted for physical function at baseline). Each new model adjusted for variables in addition to those included in the previous model (eg, social factors were added to the previous model adjusted for demographic characteristics).

Because baseline symptoms varied between the COVID-19–positive and COVID-19–negative groups, we performed a series of sensitivity analyses to evaluate the extent to which differences in the initial severity of illness may impact observed differences by COVID-19 status. First, we stratified our regression analyses based on initial COVID-19 testing location, comparing those who received their COVID-19 test in an emergency department (ED) or hospital with those who used an at-home test or received testing in an ambulatory setting (eg, a tent or drive-up site). We hypothesized that those who received testing in an ED or hospital (regardless of COVID-19 status) would be more likely to have experienced greater symptom severity. Second, because severity and outcomes have been correlated with age, we stratified the analysis by age at baseline.

## Results

Among 1000 participants included in the analysis, 722 (72.2%) had positive results for COVID-19, and 278 (27.8%) had negative results (Table 1). Of 998 participants at enrollment, 406 (40.7%) were aged 18 to 34 years, 286 (28.7%) were aged 35 to 49 years, 215 (21.5%) were aged 50 to 64 years, and 91 (9.1%) were 65 years and older. Among 972 participants, 644 (66.3%) identified as female. Of 984 participants, 833 (84.7%) identified as non-Hispanic, and 685 (70.3%) identified as White. Most participants were married or lived with a partner (503 of 977 individuals [51.5%]) and were privately insured (663 of 981 individuals [67.6%]) .

**Table 1.** Sociodemographic and Clinical Characteristics of Adults With Symptomatic Illness Who Received Positive vs Negative COVID-19 Test Results at Enrollment

Characteristic	Participants, No./total No. (%)^a^			*P* value
	Total (N = 1000)	Positive COVID-19 result (n = 722)	Negative COVID-19 result (n = 278)	
**Demographic characteristics**				
Age at enrollment, y				
18-34	406/998 (40.7)	288/721 (39.9)	118/277 (42.6)	.005
35-49	286/998 (28.7)	220/721 (30.5)	66/277 (23.8)	
50-64	215/998 (21.5)	160/721 (22.2)	55/277 (19.9)	
≥65	91/998 (9.1)	53/721 (7.4)	38/277 (13.7)	
Gender				
Female	644/972 (66.3)	464/703 (66.0)	180/269 (66.9)	.07^b^
Male	313/972 (32.2)	232/703 (33.0)	81//269 (30.1)	
Transgender, nonbinary, or other	15/972 (1.5)	7/703 (1.0)	8/269 (3.0)	
Ethnicity				
Hispanic	151/984 (15.3)	110/712 (15.4)	41/272 (15.1)	.88
Non-Hispanic	833/984 (84.7)	602/712 (84.6)	231/272 (84.9)	
Race^c^				
Asian, Native Hawaiian, or other Pacific Islander	89/974 (9.1)	52/706 (7.4)	37/268 (13.8)	.007
Black or African American	131/974 (13.4)	91/706 (12.9)	40/268 (14.9)	
White	685/974 (70.3)	508/706 (72.0)	177/268 (66.0)	
Other or multiple races	69/974 (7.1)	55/706 (7.8)	14/268 (5.2)	
Educational attainment				
Less than high school diploma	17/966 (1.8)	13/700 (1.9)	4/266 (1.5)	.70^b^
High school or GED diploma	106/966 (11.0)	78/700 (11.1)	28/266 (10.5)	
Some college but no degree	149/966 (15.4)	106/700 (15.1)	43/266 (16.2)	
College degree				
2 y	87/966 (9.0)	65/700 (9.3)	22/266 (8.3)	
4 y	290/966 (30.0)	218/700 (31.1)	72/266 (27.1)	
More than 4-y college degree	317/966 (32.8)	220/700 (31.4)	97/266 (36.5)	
Marital status				
Married or living with a partner	503/977 (51.5)	383/706 (54.2)	120/271 (44.3)	.008
Divorced, widowed, or separated	126/977 (12.9)	80/706 (11.3)	46/271 (17.0)	
Never married	348/977 (35.6)	243/706 (34.4)	105/271 (38.7)	
Annual family income before pandemic, $				
<10 000	70/979 (7.2)	44/707 (6.2)	26/272 (9.6)	<.001
10 000-35 000	132/979 (13.5)	91/707 (12.9)	41/272 (15.1)	
35 000-50 000	124/979 (12.7)	76/707 (10.7)	48/272 (17.6)	
50 000-75 000	126/979 (12.9)	100/707 (14.1)	26/272 (9.6)	
>75 000	483/979 (49.3)	369/707 (52.2)	114/272 (41.9)	
Prefer not to answer	44/979 (4.5)	27/707 (3.8)	17/272 (6.3)	
Health insurance				
Private only	663/981 (67.6)	499/709 (70.4)	164/272 (60.3)	.001
Private and public	42/981 (4.3)	25/709 (3.5)	17/272 (6.3)	
Public only	240/981 (24.5)	155/709 (21.9)	85/272 (31.3)	
None	36/981 (3.7)	30/709 (4.2)	6/272 (2.2)	
Employment status before pandemic				
Employed as essential worker or health care worker	409/978 (41.8)	309/707 (43.7)	100/271 (36.9)	.03
Employed as nonessential worker or non–health care worker	373/978 (38.1)	270/707 (38.2)	103/271 (38.0)	
Not employed	196/978 (20.0)	128/707 (18.1)	68/271 (25.1)	
**Clinical characteristics**				
Location of COVID-19 testing				
Hospital	144/993 (14.5)	101/715 (14.1)	43/278 (15.5)	<.001^b^
Emergency department	90/993 (9.1)	46/715 (6.4)	44/278 (15.8)	
Clinic including an urgent care clinic	143/993 (14.4)	104/715 (14.5)	39/278 (14.0)	
Tent or drive-up testing site	526/993 (53.0)	403/715 (56.4)	123/278 (44.2)	
At-home testing kit	15/993 (1.5)	10/715 (1.4)	5/278 (1.8)	
Other	75/993 (7.6)	51/715 (7.1)	24/278 (8.6)	
Hospitalization				
Hospitalized	76/937 (8.1)	72/665 (10.8)	4/272 (1.5)	<.001
Not hospitalized	861/937 (91.9)	593/665 (89.2)	268/272 (98.5)	
Preexisting conditions				
Asthma (moderate or severe)	137/934 (14.7)	85/665 (12.8)	52/269 (19.3)	.01
Hypertension or high blood pressure	169/934 (18.1)	109/665 (16.4)	60/269(22.3)	.03
Diabetes	67/934 (7.2)	39/665 (5.9)	28/269 (10.4)	.01
Overweight or obesity	280/934 (30.0)	194/665 (29.2)	86/269 (32.0)	.40
Emphysema or COPD	22/934 (2.4)	12/665 (1.8)	10/269 (3.7)	.08
Heart condition^d^	42/934 (4.5)	25/665 (3.8)	17/269 (6.3)	.10
Smoking^e^	49/934 (5.2)	38/665 (5.7)	11/269 (4.1)	.31
Kidney disease	14/934 (1.5)	8/665 (1.2)	6/269 (2.2)	.24^b^
Liver disease	17/934 (1.8)	11/665 (1.7)	6/269 (2.2)	.59^b^
Reported symptoms at baseline				
Systemic^f^	780/1000 (78.0)	626/722 (86.7)	154/278 (55.4)	<.001
Musculoskeletal^g^	572/1000 (57.2)	466/722 (64.5)	106/278 (38.1)	<.001
HEENT^h^	830/1000 (83.0)	657/722 (91.0)	173/278 (62.2)	<.001
Pulmonary^i^	604/1000 (60.4)	490/722 (67.9)	114/278 (41.0)	<.001
Cardiovascular^j^	273/1000 (27.3)	224/722 (31.0)	49/278 (17.6)	<.001
Gastrointestinal^k^	366/1000 (36.6)	289/722 (40.0)	77/278 (27.7)	<.001
Other	115/983 (11.7)	98/711 (13.8)	17/272 (6.3)	.001

Abbreviations: COPD, chronic obstructive pulmonary disease; GED, general educational development; HEENT, head, ears, eyes, nose, and throat.

^a^ The frequency of missingness differed across participant characteristics and ranged from 2 to 63 missing values. No systematic patterns in missingness were observed; therefore, missingness at random was assumed. All percentages and *P* values were calculated after excluding missing values.

^b^ For variables expected to have statistical significance of *P* < .05, *P* values were estimated using the Fisher exact method. Other *P* values were estimated using the χ^2^ test.

^c^ Participants self-reported their race according to the following categories: American Indian or Alaska Native; Asian, Native Hawaiian, or other Pacific Islander; Black or African American; White; other race; or more than 1 race among the available categories.

^d^ Heart conditions included coronary artery disease, heart failure, and cardiomyopathy.

^e^ Smoking was defined as current smoking of any type of tobacco, including smokeless tobacco.

^f^ Systemic symptoms included having a fever higher than 38 °C (100.4 °F), feeling hot or feverish, chills, experiencing repeated shaking with chills, and feeling more tired than usual.

^g^ Musculoskeletal symptoms included muscle aches and joint pains.

^h^ HEENT symptoms included runny nose, sore throat, decrease or change in smell, decrease or change in taste, hair loss, and headache.

^i^ Pulmonary symptoms included new cough, worsening of chronic cough, shortness of breath, and wheezing.

^j^ Cardiovascular symptoms included pain or tightness in chest and palpitations.

^k^ Gastrointestinal symptoms included nausea or vomiting, abdominal pain, and diarrhea (>3 loose or looser than normal stools within 24 hours).

As shown in Table 1, compared with participants in the COVID-19–positive group, those in the COVID-19–negative group were older and more likely to be of non-White race (eg, Black or African American); be unmarried; have lower annual family income; have public insurance; be unemployed; receive COVID-19 testing in an ED; and have a higher prevalence of moderate or severe asthma, hypertension or high blood pressure, or diabetes. No differences between groups were observed in the prevalence of the other 6 preexisting conditions (overweight or obesity, emphysema or chronic obstructive pulmonary disease, heart conditions, smoking, kidney disease, and liver disease) investigated in the univariate analyses. Participants in the COVID-19–positive group vs the COVID-19–negative group reported more symptoms at baseline (eg, head, ears, eyes, nose, and throat symptoms: 657 of 722 individuals [91.0%] vs 173 of 278 individuals [62.2%]) and were more likely to have been hospitalized for their symptomatic illness (72 of 665 individuals [10.8%] vs 4 of 272 individuals [1.5%]).

To evaluate the potential for nonresponse bias, we evaluated baseline characteristics among responders and nonresponders (ie, those unavailable for follow-up) to the 3-month survey (eTable 1 in Supplement 1). Compared with responders (n = 1000), nonresponders (n = 423) were more likely to be older, identify as Black or African American, have lower educational attainment, have lower income, be unemployed, receive their COVID-19 test in the ED or hospital, and report fewer symptoms at baseline.

**Figure 2.**
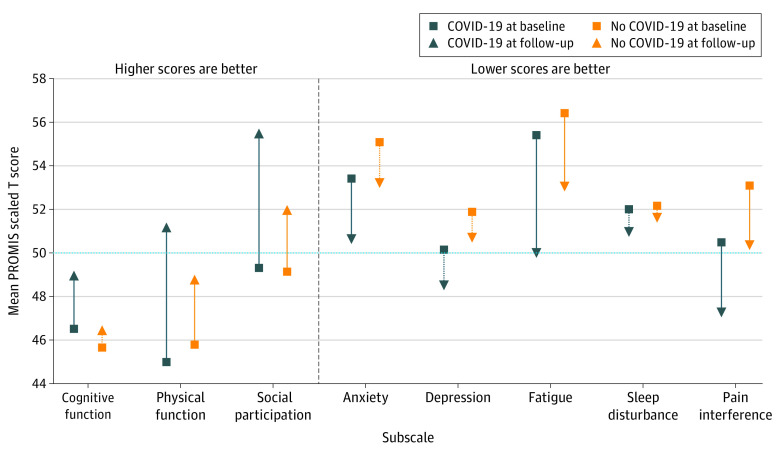
Scaled Scores on PROMIS Outcome Measures at Baseline and 3-Month Follow-up Among Participants With Positive vs Negative COVID-19 Test Results Scaled scores were based on responses to the 29-item Patient-Reported Outcomes Measurement Information System (PROMIS) survey (version 2.1) and the PROMIS Short Form–Cognitive Function 8a survey. Scores were not adjusted for demographic factors. For each domain, the mean (SD) score in the US was 50 (10) points. For cognitive function, physical, function, and social participation, higher scores are better; for all other measures, lower scores are better. Hashed lines between baseline and follow-up points indicate changes that did not meet the within-group clinically meaningful change of at least 2 points.

At baseline, participants in the COVID-19–positive group vs the COVID-19–negative group reported less anxiety (mean [SD] score, 53.4 [10.0] vs 55.1 [10.6]; *P* = .02), depression (mean [SD] score, 50.1 [9.1] vs 51.8 [9.8]; *P* = .01), pain interference (mean [SD] score, 50.5 [10.1] vs 53.0 [10.2]; *P* < .001), and pain intensity (mean [SD] score, 2.7 [2.7] vs 3.4 [2.8]; *P* < .001) (Table 2; Figure 2). In the unadjusted analyses, 459 of 709 participants (64.7%) in the COVID-19–positive group vs 182 of 270 participants (67.4%) in the COVID-19–negative group (*P* = .43) reported moderate to severe impairments across any PROMIS domain at baseline; 282 of 712 participants (39.6%) in the COVID-19–positive group vs 147 of 275 participants (53.5%) in the COVID-19–negative group (*P* < .001) reported moderate to severe impairments across any PROMIS domain at 3-month follow-up (eFigure in Supplement 1). Overall, 156 of 712 participants (21.9%) in the COVID-19–positive group and 75 of 275 participants (27.3%) in the COVID-19–negative group experienced poor mental health (ie, moderate to severe anxiety or depression) at 3-month follow-up.

**Table 2.** Unadjusted Patient-Reported Outcome Measures at Baseline and 3-Month Follow-up by COVID-19 Status at Baseline

Outcome^a^	PROMIS score, mean (SD)^b^			*P* value
	Total (N = 1000)	Positive COVID-19 result (n = 722)	Negative COVID-19 result (n = 278)	
Baseline				
Cognitive function	46.3 (11.3)	46.5 (11.4)	45.7 (11.2)	.29
Physical function	45.2 (10.3)	45.0 (10.3)	45.8 (10.1)	.29
Social participation	49.2 (12.1)	49.3 (12.4)	49.1 (11.3)	.86
Anxiety	53.9 (10.2)	53.4 (10.0)	55.1 (10.6)	.02
Depression	50.6 (9.3)	50.1 (9.1)	51.8 (9.8)	.01
Fatigue	55.7 (10.1)	55.4 (10.4)	56.4 (9.4)	.16
Sleep disturbance	52.0 (5.1)	52.0 (5.3)	52.1 (4.7)	.74
Pain interference	51.2 (10.2)	50.5 (10.1)	53.0 (10.2)	<.001
Pain intensity	2.9 (2.7)	2.7 (2.7)	3.4 (2.8)	<.001
Follow-up at 3 mo				
Cognitive function	48.2 (11.6)	48.9 (11.5)	46.4 (11.6)	.002
Physical function	50.5 (8.7)	51.2 (8.2)	48.8 (9.7)	<.001
Social participation	54.5 (11.3)	55.5 (11.0)	51.9 (11.7)	<.001
Anxiety	51.3 (10.1)	50.6 (9.8)	53.2 (10.5)	<.001
Depression	49.1 (9.2)	48.5 (8.9)	50.6 (9.7)	.001
Fatigue	50.8 (10.8)	50.0 (10.6)	53.0 (11.0)	<.001
Sleep disturbance	51.1 (4.8)	50.9 (4.7)	51.6 (4.8)	.04
Pain interference	48.2 (9.3)	47.3 (8.9)	50.3 (9.9)	<.001
Pain intensity	2.2 (2.5)	1.9 (2.4)	2.8 (2.7)	<.001
Difference between baseline and 3-mo follow-up				
Cognitive function	1.9 (9.7)	2.2 (9.9)	0.9 (9.2)	.06
Physical function	5.2 (9.7)	6.1 (9.8)	3.1 (9.2)	<.001
Social participation	5.2 (12.5)	6.1 (12.4)	2.8 (12.3)	<.001
Anxiety	−2.4 (8.9)	−2.7 (9.1)	−1.8 (8.3)	.19
Depression	−1.4 (7.7)	−1.4 (7.7)	−1.1 (7.7)	.56
Fatigue	−4.8 (9.6)	−5.3 (9.9)	−3.5 (8.8)	.01
Sleep disturbance	−0.9 (5.2)	−1.0 (5.3)	−0.5 (5.2)	.19
Pain interference	−2.9 (9.0)	−3.0 (8.8)	−2.7 (9.5)	.67
Pain intensity	−0.7 (2.4)	−0.8 (2.3)	−0.6 (2.6)	.16

Abbreviation: PROMIS, Patient-Reported Outcomes Measurement Information System.

^a^ For physical function, social participation, and cognitive function, lower scores (at baseline and follow-up) were indicative of worse outcomes; for all other domains, higher scores (at baseline and follow-up) were indicative of worse outcomes. For physical function, social participation, and cognitive function, a positive difference in scores (between baseline and follow-up) was indicative of improvement; for all other measures, a negative difference in scores (between baseline and follow-up) was indicative of improvement. Pain intensity (at baseline and follow-up) was scored from 0 (no pain) to 10 (worst imaginable pain). All other measures (at baseline and follow-up) represent a scaled T score, with a population-normed mean (SD) of 50 (10) points. For pain intensity, a within-person (ie, over time) difference of 1 or higher was considered clinically meaningful; for all other measures, an absolute difference of 2 or higher was considered clinically meaningful.

^b^ Scores were based on responses to the 29-item PROMIS survey (version 2.1) and the PROMIS Short Form–Cognitive Function 8a survey.

Compared with participants in the COVID-19–negative group, those in the COVID-19–positive group had better unadjusted improvements from baseline to 3-month follow-up for physical function (mean [SD] difference in score from baseline to follow-up, 6.1 [9.8] vs 3.1 [9.2]; difference in mean score between groups, 3.0; *P* < .001), fatigue (mean [SD] difference in score from baseline to follow-up, −5.3 [9.9] vs −3.5 [8.8]; difference in mean score between groups, −1.8; *P* = .01), and social participation (mean [SD] difference in score from baseline to follow-up, 6.1 [12.4] vs 2.8 [12.3]; difference in mean score between groups, 3.3; *P* < .001) (Table 2). After adjustment, differences in measures of well-being between baseline and follow-up were statistically and clinically better among those in the COVID-19–positive group vs the COVID-19–negative group with respect to social participation (β = 3.32; 95% CI, 1.84-4.80; *P* < .001) (Table 3). Statistically significant differences in score changes between groups were observed in other domains, but these differences were not clinically meaningful.

**Table 3.** Changes in Patient-Reported Outcome Scores Between Participants With vs Without Positive COVID-19 Test Results at Baseline

Outcome	Change in score, β (95% CI)^a^^,^^b^				
	Unadjusted model	Adjusted models			
		Demographic characteristics^c^	Social factors^d^	Health conditions^e^	Baseline T scores^f^^,^^g^
Cognitive function	1.63 (0.20 to 3.05)	1.65 (0.21 to 3.10)	1.74 (0.28 to 3.20)	1.78 (0.31 to 3.26)	1.94 (0.61 to 3.27)
Physical function	2.97 (1.56 to 4.38)	3.19 (1.76 to 4.62)	2.98 (1.54 to 4.42)	3.02 (1.57 to 4.48)	1.80 (0.72 to 2.87)
Social participation	3.82 (2.02 to 5.62)	3.91 (2.09 to 5.73)	3.68 (1.83 to 5.53)	3.59 (1.73 to 5.45)	3.32 (1.84 to 4.80)
Anxiety	−0.83 (−2.13 to 0.48)	−0.78 (−2.10 to 0.54)	−0.74 (−2.09 to 0.60)	−0.66 (−2.01 to 0.70)	−1.25 (−2.46 to −0.04)
Depression	−0.34 (−1.49 to 0.81)	−0.31 (−1.48 to 0.86)	−0.34 (−1.53 to 0.84)	−0.32 (−1.52 to 0.87)	−0.84 (−1.92 to 0.23)
Fatigue	−1.81 (−3.22 to −0.40)	−1.81 (−3.24 to −0.39)	−1.79 (−3.24 to −0.34)	−1.64 (−3.10 to −0.18)	−1.86 (−3.18 to −0.53)
Sleep disturbance	−0.68 (−1.46 to 0.09)	−0.74 (−1.53 to 0.04)	−0.86 (−1.66 to −0.07)	−0.79 (−1.58 to 0.01)	−0.65 (−1.29 to −0.01)
Pain interference	−0.25 (−1.60 to 1.11)	−0.33 (−1.70 to 1.04)	−0.33 (−1.72 to 1.06)	−0.38 (−1.78 to 1.02)	−1.35 (−2.48 to −0.23)
Pain intensity	−0.28 (−0.63 to 0.07)	−0.32 (−0.68 to 0.03)	−0.32 (−0.68 to 0.04)	−0.32 (−0.69 to 0.04)	−0.52 (−0.82 to −0.23)

Abbreviation: PROMIS, Patient-Reported Outcomes Measurement Information System.

^a^ Scores were based on responses to the 29-item PROMIS survey (version 2.1) and the cognitive function domain on the PROMIS Short Form survey.

^b^ Coefficients for participants with positive vs negative COVID-19 test results (primary exposure) are shown for all models; the difference in patient-reported outcomes (eg, physical function) over time (baseline vs follow-up) was the primary outcome. For pain intensity, a within-person (ie, over time) difference of 1 or higher was considered clinically meaningful; for all other measures, an absolute difference of 2 or higher was considered clinically meaningful. For physical function, social participation, and cognitive function, a positive difference was indicative of greater improvement among participants with positive vs negative COVID-19 test results; for all other measures, a negative difference was indicative of greater improvement. In both cases, 95% CIs inclusive of 0 indicated that differences over time for participants with positive vs negative COVID-19 test results were statistically similar at *P* ≥ .05.

^c^ Demographic characteristics included age and race.

^d^ Social factors included marital status, income, employment, and health insurance.

^e^ Health conditions included asthma, diabetes, and hypertension at 3-month follow-up.

^f^ Baseline T scores included the baseline value of each patient-reported outcome (eg, individuals’ baseline scores for physical function).

^g^ Due to a survey specification clerical error, participants were incorrectly presented with the 4 response options for the 9-item Public Health Questionnaire (ie, with 1 indicating not at all, 2 indicating several days, 3 indicating more than half of the days, and 4 indicating early every day) in place of the 5 response options for the 29-item PROMIS subdomain measuring ability to participate in social roles and activities (ie, with 1 indicating never, 2 indicating rarely, 3 indicating sometimes, 4 indicating usually, and 5 indicating always). We conducted an equivalent score mapping correction after data collection, which resulted in 4 possible scores (ie, with 1 indicating never, 2.33 indicating rarely, 3.67 indicating sometimes or usually, and 5 indicating always) to calculate the scaled T score.

Stratified sensitivity analyses revealed no significant or clinically meaningful differences in PROMIS scores over time between those in the COVID-19–positive group vs those in the COVID-19–negative group who received their COVID-19 test in the ED or hospital (ie, those who had more severe initial presentation) (eTable 2 in Supplement 1). Significantly better changes over time in several domains were observed among participants in the COVID-19–positive vs COVID-19–negative group who received testing in an ambulatory setting (eg, social participation: β = 4.16 [95% CI, 2.12-6.20]; *P* < .001; cognitive function: β = 3.33 [95% CI, 1.45-5.22]; *P* < .001). Improvements in PROMIS scores among participants in the COVID-19–positive vs COVID-19–negative group were concentrated among participants aged 18 to 34 years (eg, social participation: β = 3.90 [95% CI, 1.75-6.05]; *P* < .001; cognitive function: β = 2.91 [95% CI, 0.85-4.97]; *P* = .01), whereas participants 35 years and older experienced similar differences in PROMIS measures over time regardless of COVID-19 status.

## Discussion

In this cohort study, interim analysis of the first 1000 participants enrolled in a large, geographically diverse study revealed that a substantial proportion of individuals with positive COVID-19 results (39.6%) described moderate to severe decrements in their physical, mental, or social well-being at 3 months after symptomatic illness; however, these findings must be reviewed alongside similar results among participants with negative COVID-19 results (53.5% with moderate to severe decrements). Around the time of their acute illness, when compared with those who tested negative, participants who tested positive reported similar or slightly better well-being. As both groups progressed to 3 months after acute illness, participants in the COVID-19–positive group reported greater overall changes in their social participation compared with participants in the COVID-19–negative group. These improvements were concentrated among those who were younger and those who received testing in an ambulatory setting.

Notably, we recruited participants with acute symptoms suggestive of a first episode of COVID-19 illness, including those with and without a positive result on a COVID-19 test, which represented heterogeneous groups. The Centers for Disease Control and Prevention defines PCCs as new, ongoing, or recurrent health problems more than 4 weeks after acute SARS-CoV-2 infection; this definition is evolving and difficult to operationalize consistently across studies.^13,20^ The COVID-19–positive group in the current study contained both those who had mostly recovered after initial infection and those who might have had long COVID, for whom changes in well-being may have been different. Therefore, all results should be interpreted to reflect a more general burden of SARS-CoV-2 infection and the COVID-19 pandemic in addition to any specific burdens associated with long COVID.

The greatest observed change in well-being among participants in the COVID-19–positive group was for self-reported social participation. Given that the PROMIS subscale questions on social participation assess individuals’ ability to engage in normal activities of life (eg, “I have trouble doing all of my regular leisure activities with others”), this domain may be more salient than others when considering whether someone feels like they have long COVID and may reflect the unique experience of isolation and stigma that COVID-19–positive individuals may have endured. These findings suggest that many COVID-19–positive individuals are able to achieve well-being scores that approach the US average but do not imply that all patients with COVID-19 achieve well-being after illness. We found that 39.6% of individuals in the COVID-19–positive group reported moderate to severe impairments across any of the evaluated PROMIS well-being domains at follow-up. Future evaluations of post–COVID-19 sequelae should assess key well-being domains, including social participation, in addition to discrete symptoms, to fully capture the patient experience of long-term decrements in health and well-being. Information on social participation may specifically help to identify COVID-19 experiences for which more intense intervention and/or treatment may be required to return patients to their previous activities of daily living.

Although other studies^21,22,23,24^ have found that those who recover from acute SARS-CoV-2 infection are at increased risk of an array of mental health disorders during the subsequent year, participants in the current cohort experienced similar rates of depressive symptoms at baseline and follow-up regardless of initial COVID-19 status. The presence and persistence of poor mental health among nearly 1 in 4 participants (21.9% of the COVID-19–positive group and 27.3% of the COVID-19–negative group) may reflect a more general pandemic exposure, which participants in both groups experienced. The inclusion of a control group of participants who were exposed to the pandemic yet tested negative and the use of validated scores with prepandemic population norms were important to identifying broader pandemic impacts which may have had consequences for observed changes in well-being. For instance, similarity in observed changes in both groups may be reflective of the experience of being ill during a pandemic when access to care was hampered by pandemic restrictions, potentially slowing recovery regardless of the cause of the underlying infection. These broader pandemic societal impacts therefore call for increased attention to mental health services irrespective of SARS-CoV-2 infection status.

### Strengths and Limitations

This study has several strengths. These strengths include multicenter recruitment of patients from diverse community, ambulatory, emergency, and inpatient settings; use of concurrent controls through the recruitment of adults with symptomatic illness who tested negative for COVID-19; and prospective data collection using validated scales.

This study also has several limitations. First, although this study aimed to recruit a diverse population across the US, the requirement for access to a verifiable COVID-19 test, existing electronic health record system, and internet-enabled devices to administer study components may have biased the sample. Furthermore, those with the most severe disease may have been unable or unwilling to participate; it is possible that those too ill to participate were at higher risk of experiencing long-term symptoms after COVID-19. It is also possible that those with cognitive impairment may have been less likely to enroll.

Second, it is unclear what heterogeneous acute condition (eg, bacterial pneumonia, respiratory syncytial virus, or streptococcal pharyngitis) participants with symptomatic illness who tested negative may have been experiencing at the time of enrollment, making it difficult to hypothesize whether COVID-19–negative participants would be expected to have more or less severe patient-reported outcomes across time. Finding an appropriate comparison group for COVID-19–positive participants is difficult, and comparison with participants with symptomatic illness who test negative provides information on the ways in which infection with SARS-CoV-2 may differ from acute infection with other viruses; however, comparison with this COVID-19–negative group may underestimate the decrement in well-being compared with the general population who do not experience illness. There are many potential comparison groups that could have been used as controls in this study (eg, participants with asymptomatic illness who tested positive), and selection of a different comparator group could have yielded different results.

Third, this analysis includes participants recruited through September 2021, so findings may not be applicable to later infections involving subsequent SARS-CoV-2 variants. Fourth, COVID-19 tests may yield false-negative or false-positive results^25,26^; therefore, we cannot exclude the possibility that participants may have been misclassified (as having either a positive or negative COVID-19 test result) based on their documented test result; this misclassification could explain part of the similar change in well-being observed between the 2 groups. Fifth, our analyses only include data from participants who completed both the baseline survey and the 3-month follow-up survey; 3-month postbaseline assessment represents short-term observation of changes in well-being. Evaluating longer-term changes might provide a better understanding of the range of well-being impacts that those with COVID-19 experience.

## Conclusions

In this cohort study, SARS-CoV-2 infection was not associated with worse physical, mental, and social well-being (as measured through PROMIS scores) at 3-month follow-up compared with no SARS-CoV-2 infection among adults with symptomatic illness. Adults with acute SARS-CoV-2 infection reported substantial consequences for their well-being at baseline, with some clinically meaningful improvements at 3-month follow-up; however, a high proportion of participants in the COVID-19–positive group continued to report moderate to severe impairments in well-being at follow-up. Improvements in reported social participation domains appeared to be more substantial among participants in the COVID-19–positive group compared with the COVID-19–negative group and among younger participants and those who received testing in an ambulatory setting. These findings may reflect the impact of infection severity at presentation and emphasize the importance of comparing COVID-19–positive participants with a concurrent control group of COVID-19–negative participants as well as prepandemic population norms to identify the specific consequences of infection with SARS-CoV-2 vs the broader consequences of the COVID-19 pandemic for patient-reported outcomes.

## Notes

Supplement 1. **eAppendix.** Self-Reported Symptoms Suggestive of SARS-CoV-2 Infection **eTable 1.** Comparison of Baseline Characteristics Among Responders vs Nonresponders to the 3-Month Survey **eTable 2.** Sensitivity Analyses for Adjusted Associations Between COVID-19 Status and Difference (Between Baseline and Follow-up) in Patient-Reported Outcomes **eFigure.** Unadjusted Prevalence of Moderate to Severe Scores on PROMIS Measures at Baseline and 3-Month Follow-up for Participants With Positive vs Negative COVID-19 Test Results

Supplement 2. **Nonauthor Collaborators.** The INSPIRE Group

Supplement 3.
Data Sharing Statement

## References

[1] Hernandez-Romieu AC, Carton TW, Saydah S, . Prevalence of select new symptoms and conditions among persons aged younger than 20 years and 20 years or older at 31 to 150 days after testing positive or negative for SARS-CoV-2. JAMA Netw Open. 2022;5(2):e2147053. doi:10.1001/jamanetworkopen.2021.47053 35119459 PMC8817203

[2] Phillips S, Williams MA. Confronting our next national health disaster—long-haul COVID. N Engl J Med. 2021;385(7):577-579. doi:10.1056/NEJMp2109285 34192429

[3] Groff D, Sun A, Ssentongo AE, . Short-term and long-term rates of postacute sequelae of SARS-CoV-2 infection: a systematic review. JAMA Netw Open. 2021;4(10):e2128568. doi:10.1001/jamanetworkopen.2021.28568 34643720 PMC8515212

[4] Hernandez-Romieu AC, Leung S, Mbanya A, . Health care utilization and clinical characteristics of nonhospitalized adults in an integrated health care system 28-180 days after COVID-19 diagnosis—Georgia, May 2020–March 2021. MMWR Morb Mortal Wkly Rep. 2021;70(17):644-650. doi:10.15585/mmwr.mm7017e3 33914727 PMC8084119

[5] Hirschtick JL, Titus AR, Slocum E, . Population-based estimates of post-acute sequelae of severe acute respiratory syndrome coronavirus 2 (SARS-CoV-2) infection (PASC) prevalence and characteristics. Clin Infect Dis. 2021;73(11):2055-2064. doi:10.1093/cid/ciab408 34007978 PMC8240848

[6] Logue JK, Franko NM, McCulloch DJ, . Sequelae in adults at 6 months after COVID-19 infection. JAMA Netw Open. 2021;4(2):e210830. doi:10.1001/jamanetworkopen.2021.0830 33606031 PMC7896197

[7] Holmes EA, O’Connor RC, Perry VH, . Multidisciplinary research priorities for the COVID-19 pandemic: a call for action for mental health science. Lancet Psychiatry. 2020;7(6):547-560. doi:10.1016/S2215-0366(20)30168-1 32304649 PMC7159850

[8] Shanahan L, Steinhoff A, Bechtiger L, . Emotional distress in young adults during the COVID-19 pandemic: evidence of risk and resilience from a longitudinal cohort study. Psychol Med. 2022;52(5):824-833. doi:10.1017/S003329172000241X 32571438 PMC7338432

[9] Kämpfen F, Kohler IV, Ciancio A, Bruine de Bruin W, Maurer J, Kohler HP. Predictors of mental health during the COVID-19 pandemic in the US: role of economic concerns, health worries and social distancing. PLoS One. 2020;15(11):e0241895. doi:10.1371/journal.pone.0241895 33175894 PMC7657497

[10] Hays RD, Spritzer KL, Schalet BD, Cella D. PROMIS-29 v2.0 profile physical and mental health summary scores. Qual Life Res. 2018;27(7):1885-1891. doi:10.1007/s11136-018-1842-3 29569016 PMC5999556

[11] Cella D, Riley W, Stone A, ; PROMIS Cooperative Group. The Patient-Reported Outcomes Measurement Information System (PROMIS) developed and tested its first wave of adult self-reported health outcome item banks: 2005-2008. J Clin Epidemiol. 2010;63(11):1179-1194. doi:10.1016/j.jclinepi.2010.04.011 20685078 PMC2965562

[12] O’Laughlin KN, Thompson M, Hota B, ; INSPIRE Investigators. Study protocol for the Innovative Support for Patients with SARS-COV-2 Infections Registry (INSPIRE): a longitudinal study of the medium and long-term sequelae of SARS-CoV-2 infection. PLoS One. 2022;17(3):e0264260. doi:10.1371/journal.pone.0264260 35239680 PMC8893622

[13] Long COVID or post-COVID conditions. Centers for Disease Control and Prevention. Updated September 1, 2022. Accessed May 5, 2022. https://www.cdc.gov/coronavirus/2019-ncov/symptoms-testing/symptoms.html

[14] Magesh S, John D, Li WT, . Disparities in COVID-19 outcomes by race, ethnicity, and socioeconomic status: a systematic-review and meta-analysis. JAMA Netw Open. 2021;4(11):e2134147. doi:10.1001/jamanetworkopen.2021.34147 34762110 PMC8586903

[15] HealthMeasures. Northwestern University; 2022. Accessed October 24, 2022. https://www.healthmeasures.net/explore-measurement-systems/nih-toolbox

[16] Iverson GL, Marsh JM, Connors EJ, Terry DP. Normative reference values, reliability, and item-level symptom endorsement for the PROMIS v2.0 Cognitive Function–Short Forms 4a, 6a and 8a. Arch Clin Neuropsychol. 2021;36(7):1341-1349. doi:10.1093/arclin/acaa128 33454756

[17] HealthMeasures. PROMIS score cut points. Northwestern University; 2022. Accessed February 15, 2022. https://www.healthmeasures.net/score-and-interpret/interpret-scores/promis/promis-score-cut-points

[18] Terwee CB, Peipert JD, Chapman R, . Minimal important change (MIC): a conceptual clarification and systematic review of MIC estimates of PROMIS measures. Qual Life Res. 2021;30(10):2729-2754. doi:10.1007/s11136-021-02925-y 34247326 PMC8481206

[19] Bahreini M, Safaie A, Mirfazaelian H, Jalili M. How much change in pain score does really matter to patients? Am J Emerg Med. 2020;38(8):1641-1646. doi:10.1016/j.ajem.2019.158489 31744654

[20] Wisk LE, Nichol G, Elmore JG. Toward unbiased evaluation of postacute sequelae of SARS-CoV-2 infection: challenges and solutions for the long haul ahead. Ann Intern Med. 2022;175(5):740-743. doi:10.7326/M21-4664 35254883 PMC8906529

[21] Xie Y, Xu E, Al-Aly Z. Risks of mental health outcomes in people with COVID-19: cohort study. BMJ. 2022;376:e068993. doi:10.1136/bmj-2021-068993 35172971 PMC8847881

[22] Ueda M, Nordstrom R, Matsubayashi T. Suicide and mental health during the COVID-19 pandemic in Japan. J Public Health (Oxf). 2022;44(3):541-548. doi:10.1093/pubmed/fdab113 33855451 PMC8083330

[23] Kwong ASF, Pearson RM, Adams MJ, . Mental health before and during the COVID-19 pandemic in two longitudinal UK population cohorts. Br J Psychiatry. 2021;218(6):334-343. doi:10.1192/bjp.2020.242 33228822 PMC7844173

[24] Ping W, Zheng J, Niu X, . Evaluation of health-related quality of life using EQ-5D in China during the COVID-19 pandemic. PloS One. 2020;15(6):e0234850. doi:10.1371/journal.pone.0234850 32555642 PMC7302485

[25] Arevalo-Rodriguez I, Buitrago-Garcia D, Simancas-Racines D, . False-negative results of initial RT-PCR assays for COVID-19: a systematic review. PloS One. 2020;15(12):e0242958. doi:10.1371/journal.pone.0242958 33301459 PMC7728293

[26] Dinnes J, Deeks JJ, Berhane S, ; Cochrane COVID-19 Diagnostic Test Accuracy Group. Rapid, point-of-care antigen and molecular-based tests for diagnosis of SARS-CoV-2 infection. *Cochrane Database Syst Rev*. 2021;3(3):CD013705. 33760236 10.1002/14651858.CD013705.pub2 PMC8078597

