# Supplementary material for: Association of Initial SARS-CoV-2 Test Positivity With Patient-Reported Well-being 3 Months After a Symptomatic Illness
Source: JAMA Netw Open. 2022 Dec 1;5(12):e2244486. doi: 10.1001/jamanetworkopen.2022.44486 (PMC9716377; doi:10.1001/jamanetworkopen.2022.44486)
Supplement: Supplement 1. — eAppendix. Self-Reported Symptoms Suggestive of SARS-CoV-2 Infection eTable 1. Comparison of Baseline Characteristics Among Responders vs Nonresponders to the 3-Month Survey eTable 2. Sensitivity Analyses for Adjusted Associations Between COVID-19 Status and Difference (Between Baseline and Follow-up) in Patient-Reported Outcomes eFigure. Unadjusted Prevalence of Moderate to Severe Scores on PROMIS Measures at Baseline and 3-Month Follow-up for Participants With Positive vs Negative COVID-19 Test Results [file jamanetwopen-e2244486-s001.pdf]

Supplemental Online Content

Wisk LE, Gottlieb MA, Spatz ES, et al; INSPIRE Group. Association of initial SARS-CoV-2 test positivity with patient-reported well-being 3 months after a symptomatic illness. *JAMA Netw Open*. 2022;5(12):e2244486. doi:10.1001/jamanetworkopen.2022.44486

eAppendix. Self-Reported Symptoms Suggestive of SARS-CoV-2 Infection

eTable 1. Comparison of Baseline Characteristics Among Responders vs Nonresponders to the 3-Month Survey

eTable 2. Sensitivity Analyses for Adjusted Associations Between COVID-19 Status and Difference (Between Baseline and Follow-up) in Patient-Reported Outcomes

eFigure. Unadjusted Prevalence of Moderate to Severe Scores on PROMIS Measures at Baseline and 3-Month Follow-up for Participants With Positive vs Negative COVID-19 Test Results

This supplemental material has been provided by the authors to give readers additional information about their work.

© 2022 Wisk LE et al. *JAMA Network Open*.

eAppendix. Self-Reported Symptoms Suggestive of SARS-CoV-2 Infection

Fever
Chills
Myalgia (muscle aches)
Headache
Sore throat
Nausea or vomiting
Diarrhea
Fatigue (tiredness)
Congestion or runny nose
Cough
Shortness of breath
Difficulty breathing
New loss of taste or smell

© 2022 Wisk LE et al. *JAMA Network Open*.

eTable 1. Comparison of Baseline Characteristics Among Responders vs Nonresponders to the 3-Month Survey

	Total	Responder	Non-responder	p-value
	N (%)	N (%)	N (%)	
**Total N**	1423 (100.0%)	1000 (70.3%)	423 (29.7%)	
**Sociodemographics**				
**Age (at enrollment)**				<.001
18 to 34	544 (38.3%)	406 (40.7%)	138 (32.6%)	
35 to 49	396 (27.9%)	286 (28.7%)	110 (26.0%)	
50 to 64	333 (23.4%)	215 (21.5%)	118 (27.9%)	
65+	148 (10.4%)	91 (9.1%)	57 (13.5%)	
**Gender**				0.07
Female	903 (64.8%)	644 (66.3%)	259 (61.5%)	
Male	472 (33.9%)	313 (32.2%)	159 (37.8%)	
Transgender/Non-binary/Other	18 (1.3%)	15 (1.5%)	3 (0.7%)	
**Ethnicity**				0.16
Hispanic, Latin or Spanish origin	203 (14.5%)	151 (15.3%)	52 (12.5%)	
Not of Hispanic, Latin or Spanish origin	1,198 (85.5%)	833 (84.7%)	365 (87.5%)	
**Race**[a]				<.001
Asian/Native Hawaiian/Other Pacific Islander	106 (7.7%)	89 (9.1%)	17 (4.2%)	
Black or African American	285 (20.6%)	131 (13.4%)	154 (37.7%)	
White	895 (64.7%)	685 (70.3%)	210 (51.3%)	
Other/Multiple	97 (7.0%)	69 (7.1%)	28 (6.8%)	
**Educational Attainment**				<.001
Less than high school diploma	41 (3.0%)	17 (1.8%)	24 (5.8%)	
High school graduate or GED	222 (16.1%)	106 (11.0%)	116 (28.2%)	
2-year college degree	124 (9.0%)	87 (9.0%)	37 (9.0%)	
4-year college degree	385 (28.0%)	290 (30.0%)	95 (23.1%)	
More than 4-year college degree	390 (28.3%)	317 (32.8%)	73 (17.8%)	
Some college but did not complete degree	215 (15.6%)	149 (15.4%)	66 (16.1%)	
**Marital Status**				0.37
Married/Living with a part	702 (50.4%)	503 (51.5%)	199 (47.8%)	

© 2022 Wisk LE et al. *JAMA Network Open*.

Divorced/Widowed/Separated	189 (13.6%)	126 (12.9%)	63 (15.1%)	
Never married	502 (36.0%)	348 (35.6%)	154 (37.0%)	
**Family Income (pre-pandemic)**				<.001
Less than $10,000	129 (9.3%)	70 (7.2%)	59 (14.3%)	
$10,000 to $35,000	221 (15.9%)	132 (13.5%)	89 (21.5%)	
$35,000 to less than $50,000	173 (12.4%)	124 (12.7%)	49 (11.9%)	
$50,000 to less than $75,000	195 (14.0%)	126 (12.9%)	69 (16.7%)	
$75,000 or more	616 (44.3%)	483 (49.3%)	133 (32.2%)	
Prefer not to answer	58 (4.2%)	44 (4.5%)	14 (3.4%)	
**Health Insurance**				<.001
Private & Public	57 (4.1%)	42 (4.3%)	15 (3.6%)	
Private only	873 (62.4%)	663 (67.6%)	210 (50.4%)	
Public only	404 (28.9%)	240 (24.5%)	164 (39.3%)	
None	64 (4.6%)	36 (3.7%)	28 (6.7%)	
**Employment (pre-pandemic)**				0.001
Employed, essential or health care worker	566 (40.6%)	409 (41.8%)	157 (37.6%)	
Employed, not essential or health care worker	512 (36.7%)	373 (38.1%)	139 (33.3%)	
Not employed	317 (22.7%)	196 (20.0%)	121 (29.0%)	
**Clinical Characteristics**				
**Where received COVID test**				<.001
Hospital	250 (17.7%)	144 (14.5%)	106 (25.1%)	
Emergency department	211 (14.9%)	90 (9.1%)	121 (28.7%)	
Clinic including an Urgent Care Clinic	193 (13.6%)	143 (14.4%)	50 (11.8%)	
Tent/drive-up testing site	653 (46.1%)	526 (53.0%)	127 (30.1%)	
At home testing kit	17 (1.2%)	15 (1.5%)	2 (0.5%)	
Other	91 (6.4%)	75 (7.6%)	16 (3.8%)	
**Reported symptoms at baseline**				
Any Systemic[b]	1063 (74.7%)	780 (78.0%)	283 (66.9%)	<.001
Any Musculoskeletal[c]	774 (54.4%)	572 (57.2%)	202 (47.8%)	0.001
Any HEENT[d]	1111 (78.1%)	830 (83.0%)	281 (66.4%)	<.001
Any Pulmonary[e]	826 (58.0%)	604 (60.4%)	222 (52.5%)	0.006
Any Cardiovascular[f]	387 (27.2%)	273 (27.3%)	114 (27.0%)	0.89

© 2022 Wisk LE et al. *JAMA Network Open*.

Any Gastrointestinal[g]	515 (36.2%)	366 (36.6%)	149 (35.2%)	0.62
Any Other	141 (10.1%)	115 (11.7%)	26 (6.2%)	0.002

Proportions (%) and p-values were all calculated after excluding missingness.
For variables with cell expected values <5, p-values were estimated using Fisher exact method. Generally, the other p-values were estimated using Chi-square test.

a. Participants self-reported race according to the following verbatim categories: American Indian/Alaska Native; Asian; Native Hawaiian/Other Pacific Islander; Black or African American; Some Other Race; More than one of the above; White
b. Systemic symptoms include: fever > 100.4 F (38 C); Feeling hot or feverish; Chills; Repeated shaking with chills; More tired than usual
c. Musculoskeletal symptoms include: Muscle aches; Joint Pains
d. HEENT symptoms include: Runny Nose; Sore throat; Decrease smell or change in smell; Decrease taste or change in taste; Hair loss; Headache
e. Pulmonary symptoms include: A new cough, or worsening of chronic cough; Shortness of breath; Wheezing
f. Cardiovascular symptoms include: Pain or tightness in your chest; Palpitation
g. Gastrointestinal symptoms include: Nausea or vomiting; Abdominal pain; Diarrhea (> 3 loose/looser than normal stool/ 24 hours)

© 2022 Wisk LE et al. *JAMA Network Open*.

eTable 2. Sensitivity Analyses for Adjusted Associations Between COVID-19 Status and Difference (Between Baseline and Follow-up) in Patient-Reported Outcomes

**β (95% CI)[a]**	***Tested in ED/Hospital***	***Tested in Clinic/Drive up/At home/Other***	***Ages 18-34***	***Ages 35-49***	***Ages 50+***
**Cognitive Function**	-0.90 (-3.69, 1.88)	3.33 (1.45, 5.22)	2.91 (0.85, 4.97)	1.14 (-1.65, 3.93)	1.42 (-0.98, 3.83)
**Physical Function[c]**	0.28 (-2.16, 2.73)	3.01 (1.54, 4.48)	2.02 (0.49, 3.55)	0.40 (-1.82, 2.61)	2.49 (0.38, 4.59)
**Social Participation**	0.20 (-3.00, 3.40)	4.16 (2.12, 6.20)	3.90 (1.75, 6.05)	2.93 (-0.20, 6.05)	2.72 (-0.09, 5.53)
**Anxiety**	0.61 (-1.67, 2.90)	-2.04 (-3.77, -0.31)	-1.66 (-3.56, 0.24)	-1.04 (-3.49, 1.41)	-1.12 (-3.32, 1.08)
**Depression**	0.10 (-2.03, 2.22)	-1.49 (-3.01, 0.04)	-1.70 (-3.41, -0.00)	0.09 (-2.12, 2.29)	-0.94 (-2.81, 0.92)
**Fatigue**	-0.77 (-3.16, 1.62)	-2.48 (-4.39, -0.57)	-1.81 (-3.92, 0.30)	-0.58 (-3.06, 1.91)	-3.39 (-5.84, -0.94)
**Sleep Disturbance**	-0.40 (-1.69, 0.89)	-0.84 (-1.74, 0.06)	-0.44 (-1.39, 0.51)	-1.24 (-2.51, 0.02)	-0.67 (-1.89, 0.56)
**Pain Interference**	0.40 (-2.08, 2.89)	-1.95 (-3.51, -0.40)	-1.96 (-3.52, -0.40)	-0.16 (-2.67, 2.34)	-2.10 (-4.17, -0.02)
**Pain Intensity**	-0.17 (-0.89, 0.55)	-0.72 (-1.11, -0.33)	-1.05 (-1.48, -0.63)	0.11 (-0.52, 0.73)	-0.42 (-0.97, 0.12)

a. Coefficients for COVID + vs. COVID – participants (primary exposure) are shown for all models, where difference in patient-reported outcomes (e.g., physical function) over time (baseline vs follow-up) is the primary outcome. All models are adjusted for demographics (age and race), social factors (marital status, income, employment, health insurance), health conditions (asthma, diabetes, and hypertension), and baseline T-score. A within-person (i.e., over time) difference in pain intensity of ≥1 is considered to be clinically significant while for all other measures, an absolute difference of ≥2 is considered to be clinically significant. Positive difference for physical function, social participation, and cognitive function are indicative of greater improvement among COVID + vs. COVID – participants while negative difference in scores for all other measures are indicative of greater improvement for COVID + vs. COVID – participants. In both cases, confidence intervals inclusive of zero (0) indicate that difference over time for COVID + vs. COVID – participants are statistically similar at p≥0.05.

© 2022 Wisk LE et al. *JAMA Network Open*.

eFigure. Unadjusted Prevalence of Moderate to Severe Scores on PROMIS Measures at Baseline and 3-Month Follow-up for Participants With Positive vs Negative COVID-19 Test Results

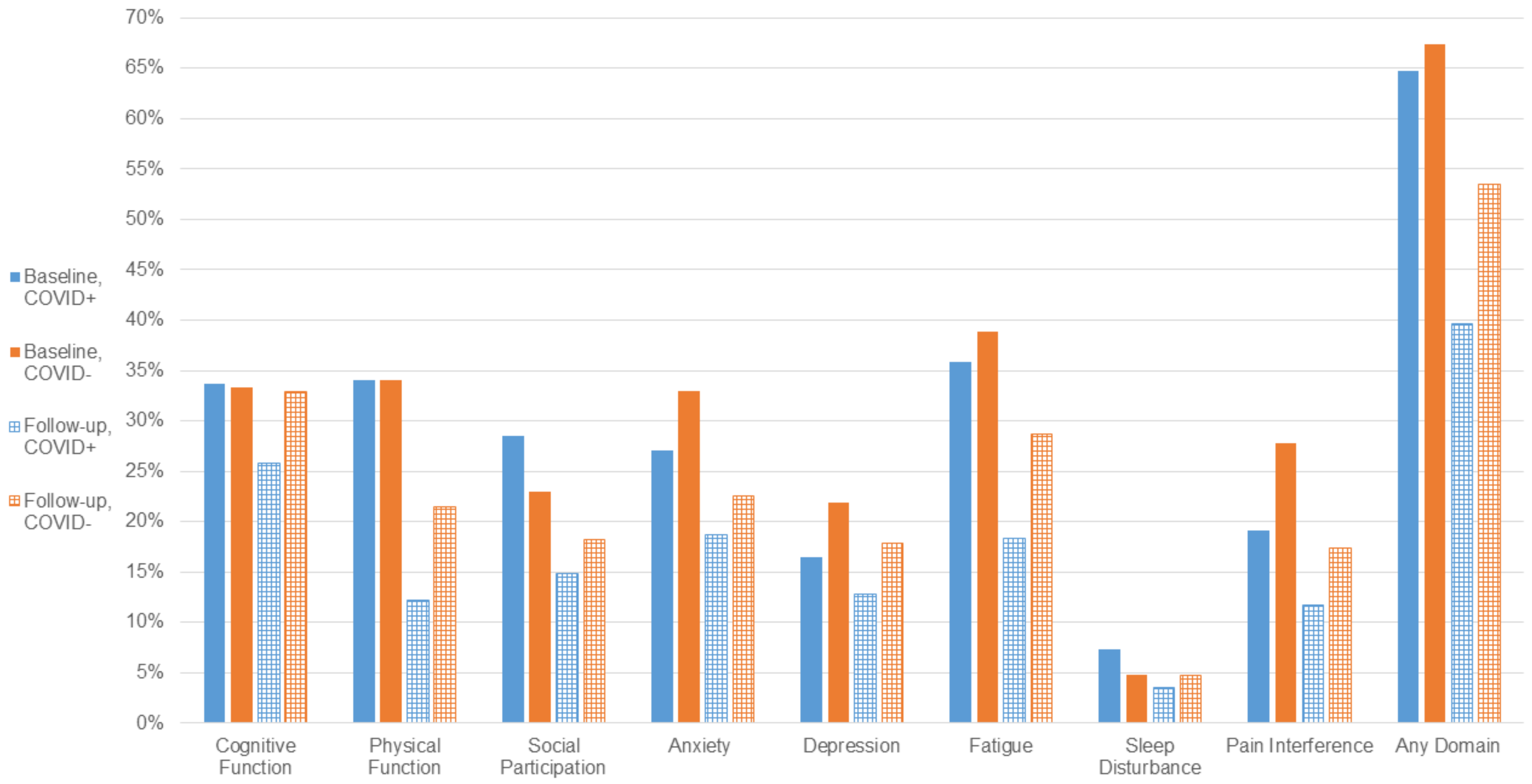

	Cognitive Function	Physical Function	Social Participation	Anxiety	Depression	Fatigue	Sleep Disturbance	Pain Interference	Any Domain
Baseline p-value	0.91	0.98	0.08	0.07	0.05	0.37	0.15	**0.003**	0.43
Follow-up p-value	**0.03**	**<.001**	0.20	0.17	**0.04**	**<.001**	0.37	**0.02**	**<.001**

PROMIS includes PROMIS-29 v2.1 and PROMIS Short Form-Cognitive Function (SF-CF 8a) questions.
Prevalence of moderate/severe scores for each PROMIS domain and across any domain are shown for COVID+ (in blue) and COVID - (in orange) participants at baseline (solid bars) and follow-up (hashed bars). P-values compare differences in the prevalence by COVID status at each time point. For physical function, social participation, and cognitive function (where higher scores are better): scores <40 represent moderate to severe impairment. For anxiety, depression, fatigue, sleep disturbance, and pain interference (where lower scores are better) scores ≥60 represent moderate to severe impairment.

© 2022 Wisk LE et al. *JAMA Network Open*.
