# Supplementary material for: Association of Initial SARS-CoV-2 Test Positivity With Patient-Reported Well-being 3 Months After a Symptomatic Illness
Source: JAMA Netw Open. 2022 Dec 1;5(12):e2244486. doi: 10.1001/jamanetworkopen.2022.44486 (PMC9716377; doi:10.1001/jamanetworkopen.2022.44486)
Supplement: Supplement 2. — Nonauthor Collaborators. The INSPIRE Group [file jamanetwopen-e2244486-s002.pdf]

Supplemental Online Content: Nonauthor Collaborators
*First name, last name, and suffix (if applicable) are required and will appear in PubMed.

*Group Name(s): The INSPIRE Group							
***First Name and Middle Initial(s)**	***Last Name**	***Suffix (eg, Jr, III)**	Academic Degrees	Institution	Location (city, state/province, country)	Role or Contribution, eg, chair, principal investigator	Group (if more than 1 Group listed in the byline) and/or Subgroup (eg, Steering Committee)
Michelle	Santangelo		MS	Rush University	Chicago, Illinois, USA	Clinical Research Coordinator	INSPIRE Group
Andrew	Ulrich		MD	Yale University	New Haven, Connecticut, USA	Co-Investigator	INSPIRE Group
Shu-Xia	Li		PhD	Yale University	New Haven, Connecticut, USA	Co-Investigator	INSPIRE Group
Jeremiah	Kinsman		MPH	Yale University	New Haven, Connecticut, USA	Project Manager	INSPIRE Group
Harlan	Krumholz		MD SM	Yale University	New Haven, Connecticut, USA	Co-Investigator	INSPIRE Group
Jocelyn	Dorney		MPH	Yale University	New Haven, Connecticut, USA	Research Associate	INSPIRE Group
Kari A	Stephens		PhD	University of Washington	Seattle, Washington, USA	Co-Principal Investigator	INSPIRE Group
Kari	Black		BA	University of Washington	Seattle, Washington, USA	Grants & Finance Manager	INSPIRE Group
Dana	Morse		RN BSN	University of Washington	Seattle, Washington, USA	Research Coordinator	INSPIRE Group
Sophie	Morse		BSc	University of Washington	Seattle, Washington, USA	Research Assistant	INSPIRE Group
Anoushka	Fernandes		BSc	University of Washington	Seattle, Washington, USA	Research Assistant	INSPIRE Group
Abhishek	Sharma		BS MS MSCS	University of Washington	Seattle, Washington, USA	Research Assistant	INSPIRE Group
Tracy	Stober		BA MA	University of Washington	Seattle, Washington, USA	Patient Representative	INSPIRE Group
Rachel E	Geyer		MPH	University of Washington	Seattle, Washington, USA	Research Coordinator	INSPIRE Group

Supplemental Online Content: Nonauthor Collaborators
*First name, last name, and suffix (if applicable) are required and will appear in PubMed.

***First Name and Middle Initial(s)**	***Last Name**	***Suffix (eg, Jr, III)**	Academic Degrees	Institution	Location (city, state/province, country)	Role or Contribution, eg, chair, principal investigator	Group (if more than 1 Group listed in the byline) and/or Subgroup (eg, Steering Committee)
Victoria	Lyon		MPH	University of Washington	Seattle, Washington, USA	Program Manager	INSPIRE Group
Karen	Adams		BA	University of Washington	Seattle, Washington, USA	Regulatory Specialist	INSPIRE Group
Michael	Willis		AS BSHS	University of Washington	Seattle, Washington, USA	Research Assistant	INSPIRE Group
Luis	Ruiz		BA	University of Washington	Seattle, Washington, USA	Research Assistant	INSPIRE Group
Jasmine	Park		N/A (in progress)	University of Washington	Seattle, Washington, USA	Research Assistant	INSPIRE Group
Kerry	Malone		BA	University of Washington	Seattle, Washington, USA	Research Assistant	INSPIRE Group
Hailey	Shughart		BA CCRP	Thomas Jefferson University	Philadelphia, Pennsylvania, USA	Research Coordinator	INSPIRE Group
Kevin W	Schaeffer		BS	Thomas Jefferson University	Philadelphia, Pennsylvania, USA	Research Coordinator	INSPIRE Group
Lindsey A	Shughart		BS	Thomas Jefferson University	Philadelphia, Pennsylvania, USA	Research Coordinator	INSPIRE Group
Abir I	Arab		BSc	Thomas Jefferson University	Philadelphia, Pennsylvania, USA	Research Coordinator	INSPIRE Group
Dylan T	Grau		BS	Thomas Jefferson University	Philadelphia, Pennsylvania, USA	Research Coordinator	INSPIRE Group
Ashini	Patel		BS	Thomas Jefferson University	Philadelphia, Pennsylvania, USA	Research Coordinator	INSPIRE Group
Phillip B	Watts		BA MM CCRP	Thomas Jefferson University	Philadelphia, Pennsylvania, USA	Research Coordinator	INSPIRE Group
Morgan	Kelly		BS	Thomas Jefferson University	Philadelphia, Pennsylvania, USA	Research Coordinator	INSPIRE Group
Alaina	Hunt		BA	Thomas Jefferson University	Philadelphia, Pennsylvania, USA	Research Coordinator	INSPIRE Group

Supplemental Online Content: Nonauthor Collaborators
*First name, last name, and suffix (if applicable) are required and will appear in PubMed.

***First Name and Middle Initial(s)**	***Last Name**	***Suffix (eg, Jr, III)**	Academic Degrees	Institution	Location (city, state/province, country)	Role or Contribution, eg, chair, principal investigator	Group (if more than 1 Group listed in the byline) and/or Subgroup (eg, Steering Committee)
Paavali	Hannikainen		BS	Thomas Jefferson University	Philadelphia, Pennsylvania, USA	Medical Student	INSPIRE Group
Melanie	Chalfin		BA	Thomas Jefferson University	Philadelphia, Pennsylvania, USA	Research Coordinator	INSPIRE Group
David	Cheng		BS	Thomas Jefferson University	Philadelphia, Pennsylvania, USA	Research Coordinator	INSPIRE Group
Jessica	Miao		BA	Thomas Jefferson University	Philadelphia, Pennsylvania, USA	Research Coordinator	INSPIRE Group
Carly	Shutty		BSN	Thomas Jefferson University	Philadelphia, Pennsylvania, USA	Clinical Research Nurse	INSPIRE Group
Summer	Chavez		DO MPH MPM	University of Texas Health Science Center at Houston	Houston, Texas, USA	Co-Investigator	INSPIRE Group
Arun	Kane		BA	University of Texas Health Science Center at Houston	Houston, Texas, USA	Research Coordinator	INSPIRE Group
Prasen	Marella		MBBS MPH	University of Texas Health Science Center at Houston	Houston, Texas, USA	Research Coordinator	INSPIRE Group
Guillermo D	Gallegos		BA	University of Texas Southwestern Medical Center	Dallas, Texas, USA	Research Coordinator	INSPIRE Group
Katherine R	Martin		MS	University of Texas Southwestern Medical Center	Dallas, Texas, USA	Research Associate	INSPIRE Group
Michelle	L'Hommedieu		PhD	University of California, Los Angeles	Los Angeles, California, USA	Project Director	INSPIRE Group
Christopher W	Chandler		BA	University of California, Los Angeles	Los Angeles, California, USA	Research Assistant	INSPIRE Group
Kate	Diaz Roldan		MPH	University of California, Los Angeles	Los Angeles, California, USA	Research Assistant	INSPIRE Group
Nicole	Villegas		BS	University of California, Los Angeles	Los Angeles, California, USA	Research Assistant	INSPIRE Group
Raul	Moreno		BA	University of California, Los Angeles	Los Angeles, California, USA	Administrative Analyst	INSPIRE Group

Supplemental Online Content: Nonauthor Collaborators
*First name, last name, and suffix (if applicable) are required and will appear in PubMed.

***First Name and Middle Initial(s)**	***Last Name**	***Suffix (eg, Jr, III)**	Academic Degrees	Institution	Location (city, state/province, country)	Role or Contribution, eg, chair, principal investigator	Group (if more than 1 Group listed in the byline) and/or Subgroup (eg, Steering Committee)
Megan	Eguchi		MPH	University of California, Los Angeles	Los Angeles, California, USA	Programmer Analyst	INSPIRE Group
Robert	Rodriguez		MD	University of California, San Francisco	San Francisco, California, USA	Co-Principal Investigator	INSPIRE Group
Robin	Kemball		MPH	University of California, San Francisco	San Francisco, California, USA	Research Manager	INSPIRE Group
Virginia	Chan		BS	University of California, San Francisco	San Francisco, California, USA	Clinical Research Coordinator	INSPIRE Group
Cecilia L	Chavez		N/A (in progress)	University of California, San Francisco	San Francisco, California, USA	Clinical Research Coordinator	INSPIRE Group
Angela	Wong		BA	University of California, San Francisco	San Francisco, California, USA	Clinical Research Coordinator	INSPIRE Group
Aron J	Hall		DVM MSPH	Centers for Disease Control and Prevention (CDC)	Atlanta, Georgia, USA	Co-Investigator	INSPIRE Group
Melissa	Briggs-Hagen		MD MPH	Centers for Disease Control and Prevention (CDC)	Atlanta, Georgia, USA	Co-Investigator	INSPIRE Group
