# Supplementary material for: Association of Initial SARS-CoV-2 Test Positivity With Patient-Reported Well-being 3 Months After a Symptomatic Illness
Source: JAMA Netw Open. 2022 Dec 1;5(12):e2244486. doi: 10.1001/jamanetworkopen.2022.44486 (PMC9716377; doi:10.1001/jamanetworkopen.2022.44486)
Supplement: Supplement 3. — Data Sharing Statement [file jamanetwopen-e2244486-s003.pdf]

Data Sharing Statement

Wisk. Association of Initial SARS-CoV-2 Test Positivity With Patient-Reported Well-being 3 Months After a Symptomatic Illness. *JAMA Netw Open*. Published December 01, 2022. doi:10.1001/jamanetworkopen.2022.44486

Data
Data available: No
